# The Use of Physical Energy-Based Therapies in the Management of Osteoarthritis

**DOI:** 10.3390/medicina62061119

**Published:** 2026-06-09

**Authors:** Marco Giuseppe Musorrofiti, Marco Bonifacio, Valerio Cipolloni, Enricomaria Mattia, Rosa Bellomo, Raoul Saggini

**Affiliations:** 1 Faculty of Exercise and Sport Sciences, Università Telematica eCampus, 22060 Novedrate, CO, Italy; marcomusorrofiti@gmail.com; 2 Fisioterapia Medica, 00053 Civitavecchia, RM, Italy; marco.bonifacio1969@gmail.com; 3 Department of Orthopaedics, A.O.U. “Vanvitelli” University Hospital, “Luigi Vanvitelli” University, Via del Sole 10, 80138 Naples, NA, Italy; 4 Morrone Center, 81100 Caserta, CE, Italy; mattia.en@virgilio.it; 5 School of Medicine, LUM University “Giuseppe Degennaro”, 70010 Casamassima, BA, Italy; bellomo@lum.it; 6 Faculty of Health Sciences, Università Telematica eCampus, 22060 Novedrate, CO, Italy; raoulsaggini@gmail.com

**Keywords:** osteoarthritis, physical modalities, extracorporeal shock wave therapy, photobiomodulation, low-level laser therapy, pulsed electromagnetic field therapy, TECAR, CRET, unloading rehabilitation, vibration therapy

## Abstract

Physical energy-based therapies are non-invasive adjunctive interventions that deliver mechanical, electromagnetic, light, or radiofrequency/thermal energy to tissues with the aim of reducing symptoms and improving tolerance of active rehabilitation. Osteoarthritis (OA) is a heterogeneous whole-joint disorder in which cartilage degeneration, subchondral bone remodeling, synovitis, peri-articular tissue dysfunction, neuromuscular impairment, and pain sensitization may interact to produce pain, stiffness, and activity restriction. As conservative therapy for OA, education, progressive therapeutic exercise, weight management when indicated, and self-management remain the core of care. Nevertheless, some patients cannot fully participate in exercise because of pain, fear of movement, load intolerance, comorbidity, or limited access to supervised rehabilitation. This narrative review synthesizes evidence published mainly between 2016 and 2026 for extracorporeal shock wave therapy (ESWT), photobiomodulation/low-level laser therapy (PBMT/LLLT), pulsed electromagnetic field therapy (PEMF), transfer energy capacitive and resistive/capacitive–resistive electric transfer (TECAR/CRET) therapy, body weight support and aquatic unloading strategies, and mechanosonic vibration therapies. The available literature suggests that ESWT and PBMT/LLLT may provide short- to mid-term pain and function benefits in selected patients with knee OA when parameters are aligned with evidence-supported dosing windows. PEMF and vibration therapies show promising but less consistent effects because protocols, devices, sham conditions, and populations vary. TECAR/CRET and unloading approaches are best interpreted as enabling tools that may reduce guarding, improve walking tolerance, or increase the quality of therapeutic exercise, rather than stand-alone disease-modifying treatments. Current national and society guidelines consistently prioritize exercise, education, and weight management; most of the modalities reviewed here are absent from guidelines or are supported only indirectly, which justifies cautious wording and individualized use. A practical application model is, therefore, time-limited and goal-oriented: identify the barrier to rehabilitation, select a modality with a plausible mechanism and published protocol, monitor pain and functional response, and discontinue the modality if it does not improve participation in active care.

## 1. Introduction

Osteoarthritis (OA) is one of the most common causes of chronic musculoskeletal pain, mobility limitation, and disability. It is now understood as a heterogeneous whole-joint disease rather than simple cartilage wear. Cartilage matrix loss, subchondral bone remodeling, osteophyte formation, meniscal and ligament changes, synovial inflammation, peri-articular muscle dysfunction, and altered movement patterns can all contribute to symptoms and progression [1].

As conservative therapy for OA, the core of care in contemporary clinical guidelines, involves education, self-management, therapeutic exercise, physical activity support, and weight management when relevant [2,3,4,5,6]. Exercise is not a single intervention; it includes strengthening, aerobic conditioning, flexibility, balance, neuromotor training, and function-specific practice. The choice of exercise should be adapted to the patient’s symptoms, comorbidities, preferences, local resources, and goals.

Physical energy-based therapies should be introduced in this context. They are not replacements for active rehabilitation and should not be presented as inherently superior to pharmacologic care, injections, transcutaneous electrical nerve stimulation (TENS), or surgery. Instead, they may have a role when a specific physical barrier prevents the patient from engaging in guideline-recommended care. For example, a short-term decrease in pain may allow gait training, strengthening, or a return to daily activity. A period of unloading may allow walking volume without provoking symptoms. A neuromuscular stimulus may help initiate exercise when quadriceps inhibition or poor confidence limits participation.

Several barriers can prevent patients from translating guideline advice into daily behavior. Pain and fear of movement may reduce physical activity, but they are not the only obstacles. Cost, access to supervised rehabilitation, transportation, comorbid disease, socioeconomic constraints, low self-efficacy, and previous negative treatment experiences also affect adherence [7,8,9]. Similarly, pharmacologic therapy and injections can be useful and, in selected patients, may produce rapid symptom relief; however, non-steroidal anti-inflammatory drugs (NSAIDs) are limited by gastrointestinal, renal, cardiovascular, and drug-interaction risks, and intra-articular injections are not suitable for repeated high-frequency use in every patient [3,4].

For these reasons, the clinically defensible question is not whether a device-based modality is ‘better’ than conventional OA care, but whether it can safely and measurably improve participation in conservative care for a defined patient phenotype. This narrative review focuses on physical energy-based interventions that are commonly encountered in rehabilitation or are increasingly discussed in clinical practice: extracorporeal shock wave therapy (ESWT), photobiomodulation/low-level laser therapy (PBMT/LLLT), pulsed electromagnetic field therapy (PEMF), transfer energy capacitive and resistive/capacitive–resistive electric transfer (TECAR/CRET), body weight support and aquatic unloading strategies, and mechanosonic vibration therapies.

The purpose of this review is to summarize plausible mechanisms, study findings, clinical protocols, safety considerations, guideline positions, and controversies. The aim is deliberately cautious: to support evidence-informed, time-limited, goal-oriented use of modalities as adjuncts to rehabilitation rather than to imply disease modification or superiority over established conservative, pharmacologic, or procedural approaches.

## 2. Methods

A structured narrative review approach was used. The search was designed to identify randomized controlled trials (RCTs), systematic reviews, meta-analyses, umbrella reviews, clinically relevant mechanistic studies, and national or society guidelines relevant to the use of non-invasive physical energy-based therapies in OA.

The primary database was PubMed/MEDLINE. Supplementary searches were performed in PubMed Central, Cochrane Library records where accessible, PEDro-indexed trial literature, Google Scholar, official clinical guideline pages, and reference lists of recent systematic reviews. The search covered studies published from 1 January 2016 to 11 February 2026, and older landmark guidelines or modality papers were retained when necessary to explain context, mechanisms, or protocol development.

Search terms combined OA location terms (‘osteoarthritis’, ‘knee osteoarthritis’, ‘hip osteoarthritis’, ‘hand osteoarthritis’, ‘thumb carpometacarpal osteoarthritis’) with intervention terms (‘extracorporeal shock wave therapy’, ‘shockwave’, ‘ESWT’, ‘photobiomodulation’, ‘low-level laser therapy’, ‘LLLT’, ‘laser therapy’, ‘pulsed electromagnetic field’, ‘PEMF’, ‘magnetotherapy’, ‘TECAR’, ‘capacitive resistive electric transfer’, ‘CRET’, ‘448 kHz radiofrequency’, ‘body-weight support’, ‘lower-body positive pressure’, ‘anti-gravity treadmill’, ‘aquatic exercise’, ‘whole-body vibration’, ‘local muscle vibration’, and ‘vibration therapy’). Guideline searches used the names of major societies and national bodies: American College of Rheumatology/Arthritis Foundation (ACR/AF), Osteoarthritis Research Society International (OARSI), European Alliance of Associations for Rheumatology (EULAR), National Institute for Health and Care Excellence (NICE), Veterans Affairs/Department of Defense (VA/DoD), and American Academy of Orthopedic Surgeons (AAOS).

Studies were eligible when they involved adults with clinically and/or radiographically diagnosed OA; evaluated a non-invasive physical energy-based therapy or unloading approach used in a rehabilitation context; and reported at least one clinical outcome such as pain intensity (visual analog scale [VAS] or numerical rating scale [NRS]), Western Ontario and McMaster Universities Osteoarthritis Index (WOMAC), Knee injury and Osteoarthritis Outcome Score (KOOS), timed up-and-go (TUG), 6 min walk test (6MWT), range of motion (ROM), patient global assessment (PGA), gait parameters, or adverse events.

Exclusion criteria were inflammatory arthritis as the dominant diagnosis; postoperative rehabilitation as the primary indication; animal or in vitro studies without clinical correlation; interventions that were invasive or primarily pharmacologic; reports with insufficient clinical outcome reporting; and duplicate publications. The reasons for exclusion at full-text level were wrong population, wrong intervention, postoperative indication, non-clinical design, non-OA diagnosis, inaccessible core outcomes, or duplicate/secondary report.

**Figure 1:**
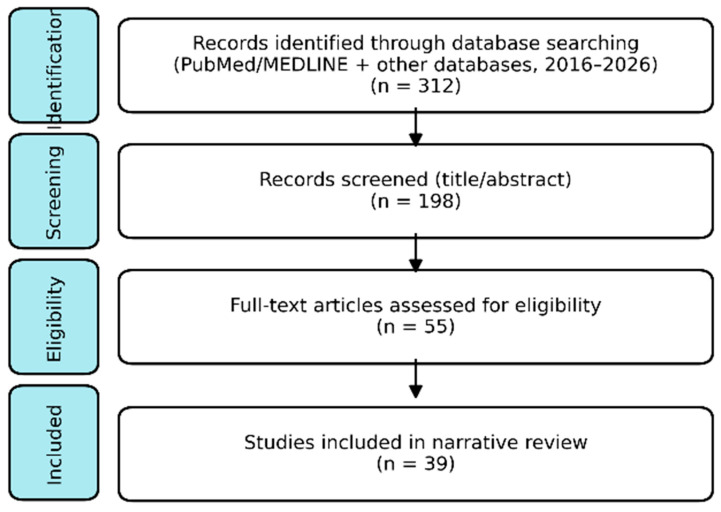
Flow diagram of evidence identification, screening, eligibility assessment, and inclusion for the narrative synthesis.

The following items were extracted when available: study design, sample size, age, sex, body mass index (BMI) or anthropometry, OA location, radiographic severity (for example Kellgren–Lawrence [KL] grade), disease duration, treatment protocol, comparator, outcomes, numerical effect estimates, adverse events, and follow-up. Because devices, dosing units, target tissues, sham conditions, and comparator interventions were heterogeneous, no new meta-analysis was performed. Numerical outcomes presented in the text and tables are taken from published trials and evidence syntheses. This limitation is discussed explicitly below. For clinical translation, protocol cues, patient model details, and guideline availability were summarized in separate tables; cohort variables were not inferred when the source reports did not provide them. The evidence identification, screening, eligibility assessment, and inclusion process is summarized in Figure 1.

## 3. Conceptual Framework: Mechanisms and Clinical Targets

OA pain is multifactorial. Articular cartilage is aneural; therefore, symptoms frequently arise from synovium, capsule, peri-articular tissues, subchondral bone, bone marrow lesions, muscle dysfunction, altered mechanics, and peripheral or central sensitization [10,11]. A physical modality is clinically meaningful only if it produces a measurable improvement in a relevant barrier: pain during movement, stiffness, load intolerance, neuromuscular inhibition, gait confidence, or adherence to active rehabilitation.

Mechanical/acoustic energy includes ESWT and mechanosonic vibration. ESWT delivers acoustic pressure waves, while vibration therapies deliver repetitive mechanical oscillations to the body or local muscles. Proposed effects include modulation of nociception, local blood flow, neuromuscular activation, proprioceptive input, and tissue remodeling signals. Mechanical unloading strategies, including lower-body positive pressure (LBPP) treadmill and aquatic exercise, do not deliver energy in the same sense as ESWT, but they alter effective gravitational load and are clinically relevant as physical enabling strategies.

Electromagnetic and light-based energy includes PBMT/LLLT and PEMF. PBMT uses red or near-infrared light to produce non-thermal photochemical effects, whereas PEMF exposes tissues to time-varying electromagnetic fields. Their proposed targets include inflammatory signaling, mitochondrial activity, microcirculation, and pain processing.

Radiofrequency/thermal energy includes TECAR/CRET and related capacitive–resistive monopolar radiofrequency approaches. These techniques generate endogenous tissue heating and may alter local circulation, tissue extensibility, pain, and guarding. Their most defensible rehabilitation role is as a pre-exercise facilitator, not as a stand-alone curative intervention.

Across all categories, two principles recur. First, effects appear to be parameter-sensitive; underdosing, excessive dosing, inadequate targeting, or poorly reported protocols can obscure benefit. Second, outcomes are most clinically valuable when symptom modulation is linked to progressive exercise and self-management.

## 4. Extracorporeal Shock Wave Therapy

### 4.1. Mechanism and Evidence

Extracorporeal shock wave therapy (ESWT) delivers acoustic pressure waves through the skin. Focused ESWT concentrates energy deeper and more precisely, whereas radial ESWT disperses energy more superficially. Proposed mechanisms in OA include modulation of inflammatory mediators, changes in nociceptor sensitivity, microvascular effects, and stimulation of remodeling signals at peri-articular and osteochondral interfaces.

The evidence signal is most developed in knee OA. A systematic review and meta-analysis of randomized trials reported that ESWT improved pain and function compared with control interventions, with a mean VAS pain difference of 1.7 cm (95% confidence interval [CI], 1.1 to 2.3) and WOMAC difference of 13.9 points (95% CI, 6.9 to 20.8) [12]. Medium energy density appeared more favorable than low or high energy density for WOMAC outcomes in that analysis [12]. An umbrella review later concluded that ESWT can improve VAS, WOMAC, Lequesne index, and ROM in knee OA, but also emphasized variability in review quality and primary trial protocols [13].

Individual trials illustrate the importance of protocol. In a dose-related radial ESWT RCT, 89 patients with knee OA were allocated to different intensity and shock number combinations or placebo; each active group received four weekly sessions, and the study concluded that moderate-intensity radial ESWT was effective, with higher energy density appearing more important than simply increasing the number of shocks [14]. In a head-to-head RCT of 44 patients, focused ESWT and radial ESWT both improved outcomes, but focused ESWT produced a greater VAS reduction at 4 weeks (−4.5 +/− 2.5 points versus −2.6 +/− 2.0 points) and greater improvements in WOMAC and 6MWT [15].

The evidence should still be interpreted cautiously. Many trials focus on knee OA, follow-up is often short, sham blinding can be difficult, and protocols differ by device type, target site, energy flux density, shock number, and treatment interval. Therefore, ESWT is best described as a potential short-term pain-bridge that may facilitate active rehabilitation in selected patients, not as a replacement for exercise, weight management, analgesic decision-making, or arthroplasty when indicated.

### 4.2. Practical Clinical Use and Safety

A practical ESWT course commonly involves three to six sessions, often weekly, with device-specific energy settings and targets. Candidate patients are usually those with symptomatic mild-to-moderate knee OA whose pain limits walking, strengthening, or stair practice. A reasonable treatment goal is achieving sufficient improvement in movement-related pain or function to progress rehabilitation within 2 to 6 weeks. Local soreness, erythema, petechiae, and bruising are usually mild and transient. Clinicians should avoid treatment over local infection, malignancy, open wounds, or areas where bleeding risk is unacceptable and should follow device-specific precautions for pregnancy, anticoagulation, and implants [5].

Photobiomodulation (PBMT), historically described as low-level laser therapy (LLLT), applies red or near-infrared light at non-thermal doses. Proposed mechanisms include absorption by mitochondrial chromophores, modulation of oxidative stress, adenosine triphosphate signaling, inflammatory pathways, and nociceptive processing. It is attractive in OA because it is non-invasive, usually painless, and can be combined with exercise.

Dose is central. A dose-specific systematic review and meta-analysis concluded that LLLT reduced pain and disability in knee OA when administered at 4 to 8 J per treatment point for wavelengths of 785 to 860 nm and 1 to 3 J per point at 904 nm [16]. This finding is important because earlier inconsistent results may reflect protocols that delivered inadequate or poorly targeted energy.

Evidence for PBMT as an adjunct to exercise is developing. A meta-analysis of older adults found statistically significant benefits of PBMT added to long-term exercise for WOMAC total score (mean difference [MD], −6.83; 95% CI, −12.30 to −1.37), WOMAC pain (MD, −2.03; 95% CI, −4.06 to −0.01), WOMAC function (MD, −5.03; 95% CI, −9.11 to −0.96), VAS/NRS pain (MD, −1.24; 95% CI, −2.43 to −0.06), and knee ROM (MD, 1.47 degrees; 95% CI, 0.07 to 2.88), while exercise endurance did not clearly improve [17].

However, PBMT remains debated. Some trials have found no incremental benefit when PBMT is added to a strengthening program, which may reflect dosing, patient selection, exercise quality, baseline severity, contextual effects, or insufficient power [18]. Therefore, PBMT should be described as a low-risk adjunct that may reduce pain and disability when evidence-supported doses are used, not as a consistently superior therapy across all OA populations [5].

Most PBMT/LLLT clinical protocols involve two to three sessions per week for 4 to 12 weeks. Reporting should include wavelength, power, energy per point, number and location of treatment points, spot size, exposure time, total energy, number of sessions, and co-interventions. Both the patient and operator should use appropriate eye protection. Direct treatment over known malignancy should be avoided; caution is warranted with photosensitivity conditions, pregnancy-related local policies, and treatment near sensitive endocrine areas. A practical placement is early in a rehabilitation session, followed immediately by strengthening, gait training, or functional practice while symptoms are reduced.

## 5. Pulsed Electromagnetic Field Therapy

Pulsed electromagnetic field therapy (PEMF), often described in clinical settings as magnetotherapy, exposes tissues to time-varying electromagnetic fields. Devices differ substantially in frequency, intensity, waveform, duty cycle, coil configuration, exposure duration, and total number of sessions. This heterogeneity is one of the major reasons the evidence remains difficult to translate.

A BMJ Open meta-analysis concluded that PEMF could alleviate pain and improve physical function in knee and hand OA, with knee OA function improvement versus sham reported as SMD −0.34 (95% CI, −0.53 to −0.14) and hand OA function improvement as SMD −1.49 (95% CI, −2.12 to −0.86); adverse events were not significantly different from sham in the trials that reported them [19]. Another systematic review and meta-analysis reported pooled improvements for pain (SMD, 0.71; 95% CI, 0.08 to 1.34), stiffness (SMD, 1.34; 95% CI, 0.45 to 2.23), and physical function (SMD, 1.52; 95% CI, 0.49 to 2.55), but again emphasized the need for higher-quality, better standardized trials [20].

Recent trial evidence remains mixed. A double-blind randomized study of 70 women with primary knee OA (mean age 59.74 +/− 9.82 years; range 40 to 80 years) added PEMF or sham PEMF to 15 physical therapy sessions over 3 weeks and reported greater VAS pain reduction and better WOMAC stiffness/function and PGA recovery ratios in the PEMF group at follow-up [21]. Conversely, a randomized trial comparing progressive resistance exercise (PRE) with or without PEMF found both approaches effective, but no clear added benefit from PEMF, suggesting that parameters and patient selection remain unresolved [22].

PEMF is generally well tolerated, but implanted electronic devices such as pacemakers, defibrillators, and neurostimulators require device-specific review or specialist advice. Pregnancy and active malignancy are commonly treated as precautionary exclusions. Clinically, PEMF should be offered only with realistic expectations, published device parameters, a planned review point after 4 to 6 weeks, and a concurrent exercise plan.

## 6. Enabling and Emerging Physical Energy Approaches

### 6.1. TECAR/CRET and Capacitive–Resistive Radiofrequency

Transfer energy capacitive and resistive (TECAR) therapy, capacitive–resistive electric transfer (CRET), and related capacitive–resistive monopolar radiofrequency techniques deliver radiofrequency energy through capacitive and/or resistive electrodes. The clinical intention is usually controlled endogenous heating, with possible effects on tissue temperature, blood flow, pain, stiffness, and guarding.

The knee OA evidence base is smaller than that for ESWT or PBMT/LLLT but includes controlled clinical studies. In a three-group randomized controlled trial of 45 patients with knee OA, 448 kHz capacitive–resistive monopolar radiofrequency was provided in eight sessions over 4 weeks with standard care. Compared with sham, active treatment improved VAS pain immediately after treatment (MD, 0.79; 95% CI, 0.29 to 1.30; effect size, 1.3), and compared with standard care alone, active treatment improved VAS pain (MD, 0.82; 95% CI, 0.32 to 1.30; effect size, 1.5) and WOMAC function (MD, 1.3; 95% CI, 0.02 to 2.6; effect size, 0.94); effects were not maintained for all outcomes at 3 months [23].

A randomized trial of CRET in knee OA reported that a 2-week program reduced pain, stiffness, and functional limitation compared with sham, and more recent clinical work has described improvements in pain, disability, and quality of life [24,25]. However, the overall evidence remains limited by small samples, device variation, sensory cues that complicate blinding, and uncertainty about whether benefits exceed non-specific heat, attention, or contextual effects.

A practical use model is therefore narrow: TECAR/CRET may be used for 10 to 20 min before exercise to reduce stiffness or guarding, then followed by strengthening, gait training, ROM work, or functional practice. The clinical test assesses whether the patient performs more repetitions, tolerates greater walking volume, or moves with better quality after treatment. If not, continuing repeated passive sessions is difficult to justify.

Safety screening should address impaired sensation, open wounds, acute infection, thrombosis, malignancy, heat intolerance, and device-specific precautions for electronic implants or metal hardware. Skin comfort and thermal sensation should be monitored throughout, especially in older adults or patients with neuropathy.

### 6.2. Body Weight Support, Lower-Body Positive Pressure, and Aquatic Unloading

Unloading approaches reduce effective joint load to permit walking, conditioning, and gait retraining with less pain. Lower-body positive pressure (LBPP) treadmills use an inflatable chamber to support a selected percentage of body weight. Aquatic exercise uses buoyancy and hydrostatic pressure to create a lower-load environment.

An RCT in 18 patients with mild-to-moderate knee OA compared LBPP walking with conventional over-ground walking for 30 min per day, 6 days per week for 2 weeks. Both groups improved WOMAC and VAS scores (LBPP WOMAC 70.25 +/− 13.93 to 40.50 +/− 11.86; VAS 3.88 +/− 0.99 to 1.63 +/− 0.52; control WOMAC 69.20 +/− 8.88 to 48.10 +/− 8.67; VAS 3.80 +/− 0.79 to 2.60 +/− 0.70; *p* < 0.001), while the LBPP group improved walking speed, stride length, and knee ROM during walking more than control [26].

A pilot crossover study in 32 people with knee OA aged over 50 found that walking at 50% body weight on an LBPP treadmill, compared with 100% body weight, acutely reduced knee pain, increased stride length, decreased cadence, and avoided the 45 min cartilage oligomeric matrix protein increase observed after full-body-weight walking [27]. These findings support unloading as a way to increase aerobic walking exposure without exacerbating pain in selected patients.

Aquatic exercise is a more accessible unloading strategy in many settings. A 2025 systematic review of randomized controlled trials in older people with OA found significant improvements in balance, stiffness, pain, and walking ability compared with non-exercise controls, while differences compared with land-based exercise were less consistent [28]. This supports aquatic exercise as a bridge to land-based loading rather than a permanent substitute for strengthening and weight-bearing function.

Unloading should always be progressed. Starting at 50% to 80% body weight may be appropriate depending on pain, gait, and comorbidity, but the goal is to increase load tolerance and transition toward functional walking, strength, and self-management. Long-term avoidance of loading can contribute to deconditioning.

### 6.3. Mechanosonic Vibration Therapies

Mechanosonic vibration therapies apply repeated mechanical oscillations through a platform, chair, bed, or local applicator. Whole-body vibration (WBV) exposes the body to platform-based vibration, whereas local muscle vibration (LMV) targets a muscle belly or tendon. Proposed effects include enhanced proprioceptive input, reflexive neuromuscular activation, altered pain thresholds, and low-impact conditioning.

A systematic review and meta-analysis of WBV in knee OA included 14 RCTs involving 559 patients; 10 studies contributed to the meta-analysis. WBV combined with strengthening exercises improved pain (SMD, 0.46; 95% CI, 0.20 to 0.71; *p* = 0.0004), WOMAC function (SMD, 0.51; 95% CI, 0.27 to 0.75; *p* < 0.0001), TUG (SMD, 0.82; 95% CI, 0.46 to 1.18; *p* < 0.00001), and several knee extensor strength outcomes, while stiffness, balance, quality of life, and knee flexor strength were not consistently improved; no adverse events were reported [29].

An umbrella review of vibration therapy in knee OA concluded that most included reviews reported favorable effects on WOMAC physical function and pain, but findings were inconsistent for disability and some performance outcomes [30]. A systematic review of LMV found promising effects on pain, stiffness, function, and knee ROM, but the number and quality of studies were limited [31]. A randomized trial of low-magnitude, variable-frequency vibration in 32 adults with moderate knee OA reported that the intervention was safe and improved pain perception and mobility compared with sham, with no adverse events reported [32].

Vibration may be useful when patients cannot tolerate higher-load strengthening or when neuromuscular inhibition is prominent. Safety screening should address fall risk, vestibular disorders, neuropathy, acute thrombosis, severe osteoporosis or fracture risk, and unstable cardiovascular disease. For frail or high-fall-risk patients, seated, supine, or local vibration is preferable over unsupported standing WBV [8].

A reviewer specifically requested for an analysis of national guidelines and major society guidelines. The most consistent finding across NICE, ACR/AF, OARSI, EULAR, VA/DoD, and AAOS guidance is that education, exercise, physical activity support, and weight management are core treatments for OA [2,3,4,5,6,33,34]. Adjunctive modalities are handled inconsistently and are often absent. This section maps whether the reviewed modalities are explicitly recommended, indirectly discussed, not addressed, or discouraged in national and society guidance. A systematic review of clinical practice guidelines similarly found broad consistency around education, exercise, and weight management priorities [35].

NICE NG226 recommends therapeutic exercise tailored to the person with OA and considers supervised exercise, education, and behavior-change approaches as part of a structured package [2]. The NICE evidence review includes electrotherapy evidence, but the guideline does not promote the modalities reviewed here as central disease management strategies. ACR/AF strongly recommends exercise and weight loss when indicated and strongly recommends against TENS for knee and/or hip OA; it does not endorse ESWT, PBMT/LLLT, PEMF, TECAR/CRET, or vibration as core therapies [3].

OARSI guidelines similarly emphasize arthritis education, structured land-based exercise, and weight management for relevant knee OA phenotypes, with pharmacologic recommendations individualized by comorbidity [4]. EULAR hip/knee recommendations emphasize exercise, information, lifestyle, weight management, assistive devices, footwear, and work participation; EULAR hand OA recommendations include education, ergonomic principles, exercises, orthoses, topical therapy, and selected pharmacologic options, but not the major device-based modalities reviewed here as standard care [5,6].

AAOS guidance for knee OA includes non-pharmacologic and pharmacologic interventions and identifies some electrotherapeutic agents as topics of evidence review; however, it encourages further study of laser treatment rather than presenting it as established core care [34]. VA/DoD guidance offers a framework for non-surgical hip and knee OA treatment that includes non-pharmacologic, pharmacologic, complementary, and surgical referral options, but it similarly prioritizes structured conservative management rather than device-based energy therapies [33].

This guideline mapping supports conservative clinical interpretation. Physical energy-based therapies may be considered optional adjuncts in carefully selected patients when they improve participation in active care, but they should not be framed as guideline-level replacements for education, exercise, weight management, appropriate analgesics, injections when indicated, or surgical referral in advanced disease. The availability of the reviewed modalities in major guidelines is summarized in Table 1.

## 7. Practical Integration into OA Rehabilitation

The most defensible clinical workflow is barrier-based. First, identify the primary barrier: movement-related pain, load intolerance, stiffness/guarding, neuromuscular inhibition, low confidence, or poor exercise adherence. Second, choose the modality that plausibly addresses that barrier. Third, pair the modality with an active rehabilitation task during the same treatment window. Fourth, reassess objective and patient-reported outcomes after a predefined short trial. An evidence summary of the reviewed modalities is presented in Table 2. Detailed protocol cues are provided in Table 3, while representative study designs and available patient model characteristics are summarized in Table 4.

**Table 2:** Evidence summary of physical energy-based modalities in osteoarthritis.

Modality	Key Study/Design Details	Numerical Results and Interpretation	Cautions
ESWT	Meta-analysis of 14 RCTs including 782 participants and 877 knees; knee OA. Dose-related radial ESWT RCT: n = 89. Focused vs. radial ESWT RCT: n = 44.	VAS pain MD 1.7 cm (95% CI, 1.1 to 2.3) and WOMAC MD 13.9 points (95% CI, 6.9 to 20.8) vs. comparators. Focused ESWT reduced VAS by −4.5 +/− 2.5 vs. radial −2.6 +/− 2.0 at 4 weeks.	Best evidence in knee OA. Protocols vary by device, energy, target, and number of shocks. Use as short-term pain-bridge to active care.
PBMT/LLLT	Dose-specific systematic review of placebo-controlled trials; adjunct-to-exercise meta-analysis in older adults.	Effective dose windows: 4–8 J/point at 785–860 nm and 1–3 J/point at 904 nm. PBMT plus exercise improved WOMAC total MD −6.83, WOMAC pain MD −2.03, WOMAC function MD −5.03, VAS/NRS MD −1.24, ROM MD +1.47 degrees.	Dose-sensitive. Some trials show no added value over strengthening. Report all laser parameters and pair with exercise.
PEMF	BMJ Open meta-analysis of knee, hand, and cervical OA; later systematic review/meta-analysis; RCT of 70 women with knee OA receiving PEMF + PT vs. sham + PT.	Knee OA function SMD −0.34 (95% CI, −0.53 to −0.14) vs. sham in one meta-analysis. Later pooled effects: pain SMD 0.71, stiffness SMD 1.34, function SMD 1.52. RCT reported better VAS, WOMAC stiffness/function, and PGA at follow-up.	Device parameters are highly heterogeneous. Some exercise-combination trials show no incremental benefit. Avoid or seek specialist advice with implanted electronic devices.
TECAR/CRET/448 kHz CRMRF	Three-group RCT in 45 knee OA patients: active CRMRF, sham, or standard care; 8 sessions over 4 weeks. Additional smaller CRET/TECAR knee OA studies.	Active CRMRF improved post-treatment VAS vs. sham (MD 0.79; 95% CI, 0.29 to 1.30; effect size 1.3) and vs. control (MD 0.82; 95% CI, 0.32 to 1.30; effect size 1.5); WOMAC function improved vs. control (MD 1.3; 95% CI, 0.02 to 2.6).	Evidence is smaller and blinding is difficult because heat is perceptible. Use as pre-exercise facilitator, not stand-alone care.
LBPP treadmill/unloading	RCT in 18 mild-to-moderate knee OA patients; LBPP walking vs. over-ground walking 30 min/day, 6 days/week for 2 weeks. Pilot crossover study in 32 adults > 50 years.	RCT: LBPP WOMAC 70.25 +/− 13.93 to 40.50 +/− 11.86; VAS 3.88 +/− 0.99 to 1.63 +/− 0.52. Control also improved, but LBPP improved walking speed, stride length, and knee ROM more. 50% body-weight walking reduced acute pain and improved gait vs. 100% body weight.	Requires equipment and progression to full loading. Not a substitute for strengthening.
Aquatic exercise	2025 systematic review of RCTs in older people with OA; included varied OA locations and intervention protocols.	Improved balance, stiffness, pain, and walking ability compared with non-exercise controls (*p* < 0.05). Differences vs. land-based exercise were less consistent.	Use low-load bridge when land exercise is poorly tolerated. Access, pool safety, skin/infection issues, and cardiopulmonary tolerance must be considered.
WBV/LMV/vibrotherapy	WBV meta-analysis: 14 RCTs, 559 knee OA patients. LMV systematic review. Low-magnitude vibration RCT: n = 32.	WBV + strengthening improved pain SMD 0.46, WOMAC function SMD 0.51, TUG SMD 0.82, and knee extensor strength outcomes. LMV improved pain, stiffness, function, and ROM in limited studies. Low-magnitude vibration was safe and improved pain perception/mobility.	Evidence promising but inconsistent. Screen fall risk, vestibular disorders, neuropathy, thrombosis, and cardiovascular instability.

**Table 3:** Practical clinical protocol cues for adjunctive use.

Clinical Barrier	Possible Modality	Typical Protocol Cues	Outcome Target and Stop Rule
Movement-related pain preventing exercise	ESWT; PBMT/LLLT	ESWT commonly 3–6 sessions, often weekly; PBMT/LLLT commonly 2–3 sessions/week for 4–12 weeks. Use published dose windows and device parameters.	Target: lower pain during gait/strengthening and improved walking or training volume. Stop if no functional gain after a short, predefined trial.
Load intolerance or flare-prone walking	LBPP treadmill; aquatic exercise	Start at tolerable unloading, often 50–80% body weight for LBPP, then progress load. Aquatic exercise 2–3 sessions/week with transition to land tasks.	Target: increased walking volume without symptom flare, improved gait confidence, and transition to land-based strengthening.
Stiffness, guarding, poor tolerance of ROM or strengthening	TECAR/CRET	10–20 min before exercise; monitor thermal comfort and skin. Follow immediately with mobility, strength, gait, or task practice.	Target: better movement quality, ROM, repetitions, or walking tolerance during the same session. Stop if it becomes passive care without measurable benefit.
Neuromuscular inhibition or low-impact conditioning need	WBV, LMV, seated/supine vibrotherapy	Short, supervised bouts; choose standing WBV only if fall risk is low. Consider local or supine delivery for frail patients.	Target: better quadriceps activation, sit-to-stand, TUG, or exercise adherence. Stop if dizziness, pain flare, or no functional progression occurs.
Preference for non-pharmacologic adjunct with low sensory burden	PEMF	Use device-specific protocol from published studies; often repeated sessions over 3–8 weeks. Confirm implant precautions.	Target: incremental improvement in pain/function above exercise program. Stop if no change after 4–6 weeks.

**Table 4:** Representative study design and patient model details requested by reviewers; variables are reported only when available in the source reports and are not inferred.

Study/Reference	Design and Population	Protocol and Comparator	Outcomes Reported
Zhang et al. [14]	2 × 2 factorial RCT; 89 patients with knee OA.	Radial ESWT with different intensity (0.12 or 0.24 mJ/mm^2^) and shock number (2000 or 4000 impulses), 4 weekly sessions; placebo control.	Pain score; dose–response suggested higher density more effective than higher shock number.
Ko et al. [15]	Randomized comparative study; 44 patients with knee OA; focused ESWT (n = 22) vs. radial ESWT (n = 22).	Comparable treatment parameters over 4 weeks; assessor-blinded but no true sham control.	VAS, WOMAC, ROM, 6MWT. VAS change at 4 weeks: focused −4.5 +/− 2.5 vs. radial −2.6 +/− 2.0.
Stausholm et al. [16]	Systematic review/meta-analysis of randomized placebo-controlled trials in knee OA.	Dose-stratified LLLT/PBMT by wavelength and energy per point.	Pain and disability improved at 4–8 J/point for 785–860 nm and 1–3 J/point for 904 nm.
Li et al. [17]	Meta-analysis of RCTs in older adults with knee OA receiving PBMT with exercise.	PBMT plus long-term exercise vs. exercise/control conditions.	WOMAC total, pain, function, VAS/NRS, ROM. Significant benefits for pain/function, not exercise endurance.
Hashemi et al. [21]	Double-blind RCT; 70 female patients with primary knee OA; mean age 59.74 +/− 9.82 years, range 40–80.	Both groups received 15 PT sessions over 3 weeks; active PEMF vs. sham PEMF.	VAS, WOMAC stiffness/function, PGA. PEMF group had greater pain reduction and physical improvement.
Kumaran and Watson [23]	Three-group RCT; 45 knee OA patients from physiotherapy waiting list; participant blinding and intention-to-treat analysis.	Active 448 kHz CRMRF vs. sham CRMRF vs. standard care; 8 sessions over 4 weeks plus follow-ups to week 16.	VAS, WOMAC, TUG, ROM. Short-term VAS and WOMAC benefits; no meaningful TUG/ROM differences and no sustained 3-month differences.
Chen et al. [26]	RCT; 18 mild-to-moderate knee OA patients.	LBPP treadmill walking vs. conventional over-ground walking, 30 min/day, 6 days/week for 2 weeks.	WOMAC, VAS, 3D gait analysis. Both groups improved symptoms; LBPP improved walking speed, stride length, knee ROM more.
Qiu et al. [29]	Systematic review/meta-analysis; 14 RCTs, 559 knee OA patients.	WBV with strengthening exercise vs. strengthening or other controls; 4–24-week interventions.	Pain, WOMAC function, TUG, extensor strength improved; stiffness, balance, quality of life, and flexor strength not consistently improved.
Pasterczyk-Szczurek et al. [32]	Randomized controlled trial; 32 adults with moderate knee OA.	Low-magnitude, variable-frequency vibration vs. sham therapy.	Pain perception and mobility improved; no adverse events reported.

Examples include ESWT or PBMT/LLLT when pain prevents gait training or strengthening; LBPP treadmill or aquatic exercise when walking volume is limited by load intolerance; TECAR/CRET when stiffness and guarding prevent quality exercise; and vibration when low-impact neuromuscular priming is required. Outcomes should include at least one symptom measure (VAS/NRS or WOMAC pain) and one functional measure (walking tolerance, TUG, sit-to-stand repetitions, 6MWT, KOOS function, or step count).

A practical stop rule is essential. If a modality fails to produce a meaningful improvement in training quality, walking tolerance, pain during exercise, or functional progression after a short trial, it should be discontinued. This prevents passive care dependency, reduces cost, and keeps treatment aligned with guideline principles.

## 8. Evidence Limitations and Future Research

The major limitation of this evidence base is heterogeneity. Devices differ by energy output, frequency, waveform, dose unit, electrode or probe placement, anatomical target, session number, treatment interval, and co-interventions. Outcomes and follow-up intervals vary, and many trials are small. Sensory cues such as heat, pressure, sound, or vibration make sham control difficult and may amplify contextual effects.

For these reasons, a new meta-analysis was not performed in this narrative review. Pooling would risk combining clinically dissimilar protocols and producing a misleading summary estimate. Instead, this review presents numerical estimates from existing meta-analyses and RCTs, highlights the direction and size of effects where available, and emphasizes protocol transparency.

Future studies should report complete device parameters, standardized OA phenotype information, KL grade, symptom duration, BMI, comorbidity, pain sensitization features, synovitis or bone marrow lesion status where available, co-interventions, adherence, adverse events, and cost-effectiveness. Pragmatic trials should test whether modalities improve exercise participation, not whether they merely reduce pain immediately after treatment. Longer follow-up is needed to determine whether short-term gains translate into sustained physical activity and reduced disability.

## 9. Conclusions

Physical energy-based therapies can be considered adjunctive enabling tools in OA management when used with cautious expectations and clear functional goals. ESWT and PBMT/LLLT have the most consistent evidence for short- to mid-term symptom improvement in knee OA when evidence-supported parameters are used. PEMF and vibration therapies show possible benefits but remain limited by heterogeneity and inconsistent protocols. TECAR/CRET and unloading strategies are promising mainly as pre-exercise or load-management facilitators. Guidelines from major societies and national bodies continue to prioritize education, therapeutic exercise, physical activity, and weight management; therefore, modalities should not be presented as replacements for guideline-based conservative care. The best clinical use is individualized, time-limited, and outcome-driven: select a modality to overcome a specific barrier, document parameters, monitor response, integrate active rehabilitation, and discontinue when it does not improve function or participation.

## Figures and Tables

**Table 1:** Availability of reviewed modalities in major guidelines.

Guideline	Core Recommendations Relevant to This Review	Position on Physical Energy Modalities	Implication for Manuscript Wording
NICE NG226 (2022) [2]	Offer tailored therapeutic exercise; consider supervised exercise, education, and behavior-change approaches.	Electrotherapy evidence was reviewed, but ESWT, PBMT/LLLT, PEMF, TECAR/CRET, and vibration are not presented as core OA treatments.	Modalities should be adjunctive and goal-oriented; exercise remains core conservative therapy.
ACR/AF hand, hip, knee OA guideline (2019/2020) [3]	Strong recommendations include exercise, weight loss when indicated, self-management, tai chi, selected braces/orthoses, NSAIDs, and intra-articular glucocorticoids for knee OA.	Strong recommendation against TENS for knee/hip OA; no core recommendation for ESWT, PBMT/LLLT, PEMF, TECAR/CRET, or vibration.	Avoid language implying superiority over injections or TENS; describe evidence as evolving and modality specific.
OARSI knee, hip, polyarticular OA guideline (2019) [4]	Arthritis education and structured land-based exercise, with dietary weight management for relevant knee OA phenotypes.	Adjunctive physical/electrotherapeutic modalities are not central core treatments; recommendations vary by phenotype and comorbidity.	Use individualized decision-making and avoid universal claims.
EULAR hip/knee and hand OA recommendations [5,6]	Exercise, information, lifestyle, weight management, assistive devices, orthoses, and work participation; hand OA includes ergonomic principles and hand exercises.	Device-based energy modalities are not generally guideline-level core care.	Position modalities as optional enablers of the recommended active pathway.
VA/DoD hip and knee OA guideline (2020) [33]	Structured evaluation and diagnosis; non-pharmacologic, pharmacologic, complementary, and referral options.	Does not elevate ESWT, PBMT/LLLT, PEMF, TECAR/CRET, or vibration to core treatment status.	Use shared decision-making and outcome monitoring.
AAOS knee OA non-arthroplasty guideline (2021) [34]	Covers adult knee OA non-arthroplasty management across non-pharmacologic, pharmacologic, and procedural options.	Physical/electrotherapeutic agents were included in scope; further study of laser treatment was encouraged.	Laser/PBMT should be described as promising but not definitive.

## Data Availability

No new data were created or analyzed in this study. Data sharing is not applicable to this article.

## Abbreviations

AAOS, American Academy of Orthopedic Surgeons; ACR/AF, American College of Rheumatology/Arthritis Foundation; BMI, body mass index; CI, confidence interval; CRET, capacitive–resistive electric transfer; ESWT, extracorporeal shock wave therapy; EULAR, European Alliance of Associations for Rheumatology; KL, Kellgren–Lawrence; KOOS, Knee injury and Osteoarthritis Outcome Score; LBPP, lower-body positive pressure; LLLT, low-level laser therapy; LMV, local muscle vibration; MD, mean difference; NICE, National Institute for Health and Care Excellence; NRS, numerical rating scale; NSAID, non-steroidal anti-inflammatory drug; OA, osteoarthritis; OARSI, Osteoarthritis Research Society International; PBMT, photobiomodulation; PEMF, pulsed electromagnetic field therapy; PGA, patient global assessment; PRE, progressive resistance exercise; RCT, randomized controlled trial; ROM, range of motion; SMD, standardized mean difference; TECAR, transfer energy capacitive and resistive; TENS, transcutaneous electrical nerve stimulation; TUG, timed up-and-go; VA/DoD, Veterans Affairs/Department of Defense; VAS, visual analog scale; WBV, whole-body vibration; WOMAC, Western Ontario and McMaster Universities Osteoarthritis Index; 6MWT, 6 min walk test.
